# Comparative assessment of motion averaged free-breathing or breath-held cardiac magnetic resonance imaging protocols in a porcine myocardial infarction model

**DOI:** 10.1038/s41598-022-07566-w

**Published:** 2022-03-08

**Authors:** Dinesh Selvakumar, Tejas Deshmukh, Sheryl L. Foster, Naeim N. Sanaei, Anthea L. L. Min, Stuart M. Grieve, Faraz Pathan, James J. H. Chong

**Affiliations:** 1 Centre for Heart Research, The Westmead Institute for Medical Research, The University of Sydney, 176 Hawkesbury Road, Westmead, Sydney, NSW 2145 Australia; 2 Department of Cardiology, Westmead Hospital, Westmead, NSW 2145 Australia; 3 Sydney School of Health Sciences, Faculty of Medicine and Health, The University of Sydney, Sydney, NSW 2006 Australia; 4 Department of Radiology, Westmead Hospital, Westmead, NSW 2145 Australia; 5 Imaging and Phenotyping Laboratory, Faculty of Medicine and Health, Charles Perkins Centre, The University of Sydney, Sydney, NSW 2006 Australia; 6 Department of Radiology, Royal Prince Alfred Hospital, Camperdown, NSW 2006 Australia; 7 Nepean Clinical School of Medicine, Charles Perkin Centre Nepean, University of Sydney, Kingswood, NSW 2747 Australia; 8 Department of Cardiology, Nepean Hospital, Kingswood, NSW 2747 Australia

**Keywords:** Preclinical research, Cardiology, Magnetic resonance imaging

## Abstract

Breath-held (BH) cardiac magnetic resonance imaging (CMR) is the gold standard for volumetric quantification. However, large animals for pre-clinical research are unable to voluntarily breath-hold, necessitating general anaesthesia and mechanical ventilation, increasing research costs and affecting cardiovascular physiology. Conducting CMR in lightly sedated, free-breathing (FB) animal subjects is an alternative strategy which can overcome these constraints, however, may result in poorer image quality due to breathing motion artefact. We sought to assess the reproducibility of CMR metrics between FB and BH CMR in a porcine model of ischaemic cardiomyopathy. FB or BH CMR was performed in 38 porcine subjects following percutaneous induction of myocardial infarction. Analysis was performed by two independent, blinded observers according to standard reporting guidelines. Subjective and objective image quality was significantly improved in the BH cohort (image quality score: 3.9/5 vs. 2.4/5; *p* < 0.0001 and myocardium:blood pool intensity ratio: 2.6–3.3 vs. 1.9–2.3; *p* < 0.001), along with scan acquisition time (4 min 06 s ± 1 min 55 s vs. 8 min 53 s ± 2 min 39 s; *p* < 0.000). Intra- and inter-observer reproducibility of volumetric analysis was substantially improved in BH scans (correlation coefficients: 0.94–0.99 vs. 0.76–0.91; coefficients of variation: < 5% in BH and > 5% in FB; Bland–Altman limits of agreement: < 10 in BH and > 10 in FB). Interstudy variation between approaches was used to calculate sample sizes, with BH CMR resulting in greater than 85% reduction in animal numbers required to show clinically significant treatment effects. In summary, BH porcine CMR produces superior image quality, shorter scan acquisition, greater reproducibility, and requires smaller sample sizes for pre-clinical trials as compared to FB acquisition.

## Introduction

Ischaemic heart disease is the leading cause of death and disability worldwide^1^, and there is a dire need for novel cardioprotective or regenerative strategies. Unfortunately, the translation of new therapies from the laboratory to the clinical setting has largely been disappointing^2^. Though multifactorial, part of this failure can be attributed to inadequately designed and powered pre-clinical studies, in which efficacy endpoints have either been poorly chosen or inadequately analysed^2,3^. Accurate and reproducible assessment of cardiac indices is paramount in gauging the efficacy of a new therapeutic agent, with cardiac magnetic resonance imaging (CMR) the current gold standard for volumetric quantification^4–6^. However, CMR image quality can be affected by breathing artefact^7^, and since animal subjects are unable to voluntarily breath-hold, general anaesthesia and mechanical ventilation are often required^8^. This requires specialist personnel and equipment, increasing research costs. Furthermore, anaesthesia affects cardiovascular physiology, potentially confounding results and increasing procedural risks in the acute setting post myocardial infarction^9^. An alternative approach involves only lightly sedating animal subjects, allowing for spontaneous, free-breathing (FB) throughout the duration of the scan, a strategy increasingly used in vulnerable human patients who are unable to breath-hold (BH)^10^ and also employed in large animal research^11–15^. FB acquisition negates the need for mechanical ventilation and cardio-depressant maintenance anaesthesia, however, introduces breathing artefact which may impair the integrity of acquired data.

In this study, we sought to compare the image quality and reproducibility of common CMR metrics between FB and BH scans in a porcine model of ischaemic cardiomyopathy.

## Results

### Subject characteristics

A total of 38 animals were imaged, with 0 cases aborted due to difficulty obtaining gating for imaging. Five animals died during or soon after myocardial infarct creation and did not undergo CMR. Animals included in analysis weighed 32.6 ± 8.3 kg, and CMR was performed 16.2 ± 12.2 days post myocardial infarction, with no significant difference between groups (Table 1). The time taken to acquire the cine short axis stack was significantly shorter in the BH as compared to the FB group (4 min 06 s ± 1 min 55 s vs. 8 min 53 s ± 2 min 39 s; *p* < 0.0001). All but one animal were in sinus rhythm for the duration of the scan. Mean left and right ventricular ejection fractions were 43% and 51% respectively.

**Table 1:** Clinical characteristics and CMR findings.

	All subjects (n = 38)	Free-breathing subjects (n = 19)	Breath-held subjects (n = 19)	*p* Value
**Clinical characteristics**				
Weight	32.6 ± 8.3	33.5 ± 8.0	31.7 ± 8.8	0.44
Day post MI	16.2 ± 12.2	15.2 ± 10.8	17.2 ± 13.6	0.58
Treated	12	7	5	
Control	26	12	14	
Cine SAX acquisition duration (min:s)	6:27 ± 3:22	8:53 ± 2:39	4:06 ± 1:55	< 0.0001
**Left ventricle**				
EDV	98.9 ± 25.0	99.1 ± 24.1	98.0 ± 26.9	0.98
ESV	56.9 ± 19.9	53.8 ± 18.4	60.3 ± 20.3	0.39
EF	42.0 ± 14.4	46.0 + 10.7	39.6 ± 9.7	0.06
Myocardial mass	51.0 ± 14.3	49.3 ± 12.5	52.0 ± 16.0	0.47
**Right ventricle**				
EDV	77.1 ± 21.3	71.6 ± 18.0	82.7 ± 23.3	0.24
ESV	37.6 ± 11.1	37.3 ± 12.1	37.8 ± 10.3	0.91
EF	50.9 ± 8.5	48.1 ± 8.4	53.6 ± 7.7	0.04

*CMR* cardiac magnetic resonance, *EF* ejection fraction, *EDV* end-diastolic volume, *ESV* end-systolic volume, *MI* myocardial infarction, *SAX* short axis stack.

Data presented as mean ± standard deviation.

### Image quality

**Figure 1:**
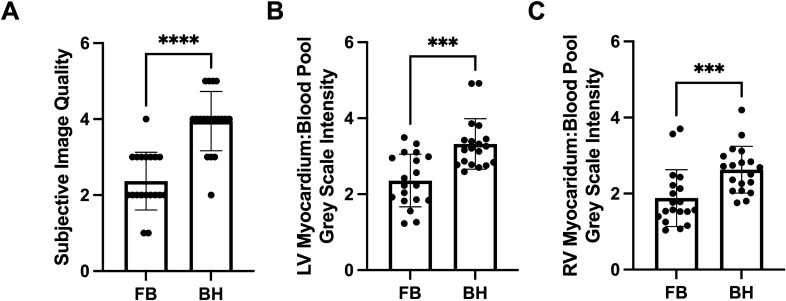
Subjective and objective assessment of image quality. (**A**) Mean image quality score for breath-held and free-breathing scans based on clarity of endocardial borders and ease of volume analysis, as subjectively assessed by two independent, blinded observers (1 = uninterpretable, 2 = poor, 3 = acceptable, 4 = good, 5 = very good). (**B**) Left ventricular myocardium to blood pool image intensity ratio. (**C**) Right ventricular myocardium to blood pool image intensity ratio. ***p < 0.001, ****p < 0.0001. *BH* breath-held scan, *FB* free-breathing scan, *LV* left ventricle, *RV* right ventricle.

Image quality was significantly improved by subjective assessment by both observers in the BH group as compared to the FB group (image quality score: 3.9 vs. 2.4; *p* < 0.0001) (Fig. 1). Only one BH scan was rated as having below acceptable image quality, and on review it was noted that this scan was complicated by an arrhythmia at the time of image acquisition. Only a small portion of FB scans had above acceptable ratings, and two scans were rated as uninterpretable by both observers.

Objective image quality was assessed by calculating the ratio of image intensity between myocardium and blood pool in both the left and right ventricular chambers^16^. This assessment showed a greater image intensity ratio in BH scans as compared to FB scans (LV ratio: 3.3 vs. 2.3; *p* < 0.001, RV ratio: 2.6 vs. 1.9; *p* < 0.001).

### Intra-observer variability

The repeated measurement of each parameter by a single observer showed excellent correlation for the BH group (r = 0.94–0.99), but a more variable series of results and generally reduced correlation was observed for the FB group (r = 0.76–0.91). All other indices of reproducibility also favoured the BH cohort (Table 2), with coefficients of variation < 5% for LVEF and RVEF in BH scans but > 5% in FB scans, mean absolute differences < 3 in BH scans but > 3 in FB scans, and Bland–Altman limits of agreements for LVEF and RVEF < 10 in BH but > 10 in FB scans.

**Table 2:** Intra-observer variability.

Parameter	Modality	Correlation co-efficient (r)	Mean absolute difference	SD of difference	CV (%)	Width of BA-LOA
LVEF	FB	0.90	3.72	4.98	7.45	12.73
	BH	0.99	1.28	1.50	2.70	3.20
RVEF	FB	0.76	3.84	6.00	7.82	15.05
	BH	0.94	1.85	2.96	4.24	7.26
LV mass	FB	0.91	5.24	5.53	9.11	16.20
	BH	0.97	4.21	3.41	6.56	11.43

*BA-LOA* Bland–Altman limit of agreement, *BH* breath-held, *CV* co-efficient of variation, *FB* free-breathing, *LV* left ventricle, *LVEF* left ventricular ejection fraction, *RVEF* right ventricular ejection fraction, *SD* standard deviation.

**Figure 2:**
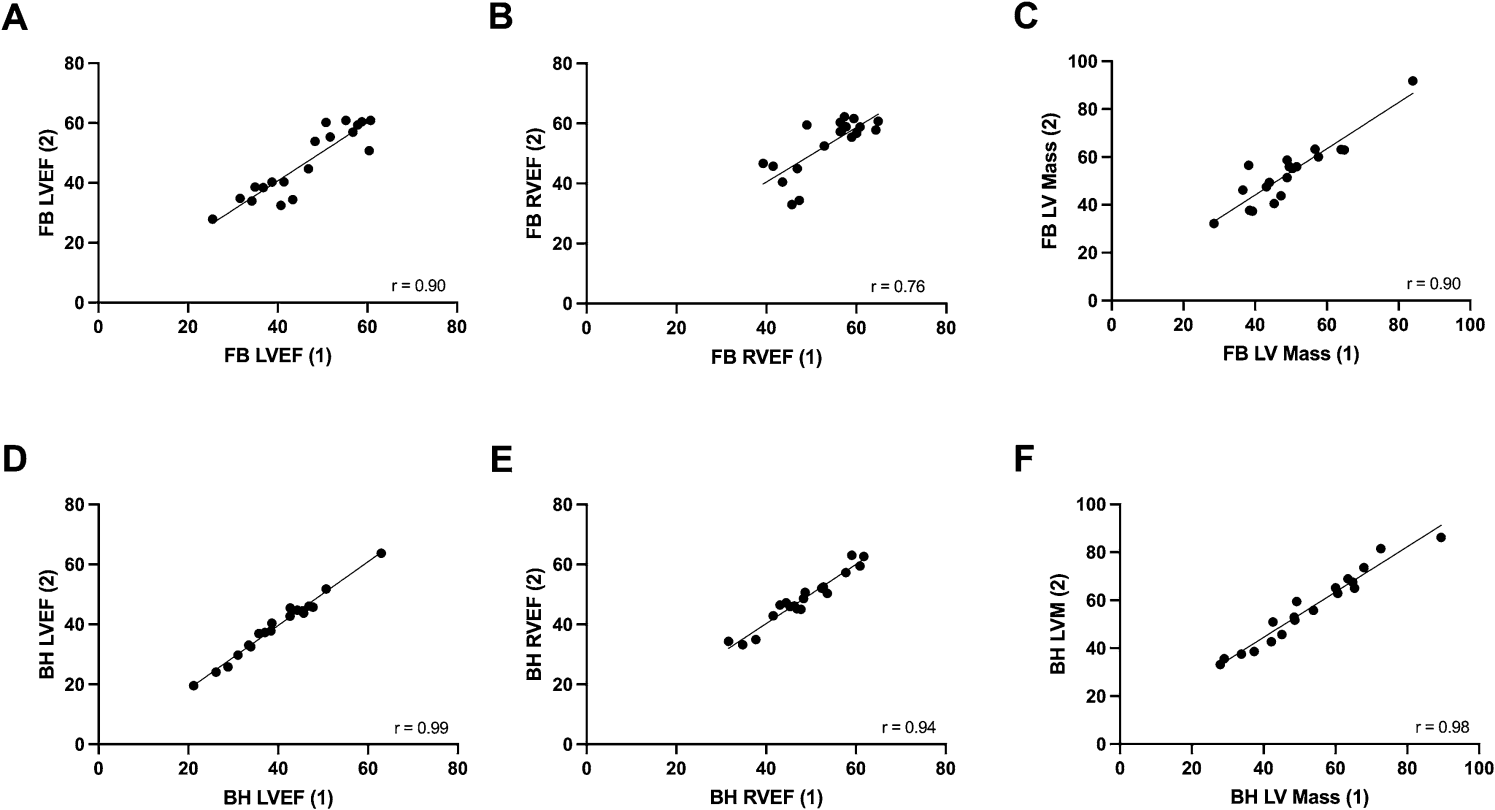
Intra-observer correlation scatter plots. (**A**) Assessment of LVEF in FB scans. (**B**) Assessment of RVEF in FB scans. (**C**) Assessment of LV Mass in FB scans. (**D**) Assessment of LVEF in BH scans. (**E**) Assessment of RVEF in BH scans. (**F**) Assessment of LV mass in BH scans. (1) = Primary observer’s first set of measurements; (2) = Primary observer’s second set of measurements. *BH* breath-held scan, *FB* free-breathing scan, *LV* left ventricle, *LVEF* left ventricle ejection fraction, *r* Pearson’s correlation co-efficient, *RVEF* right ventricle ejection fraction.

**Figure 3:**
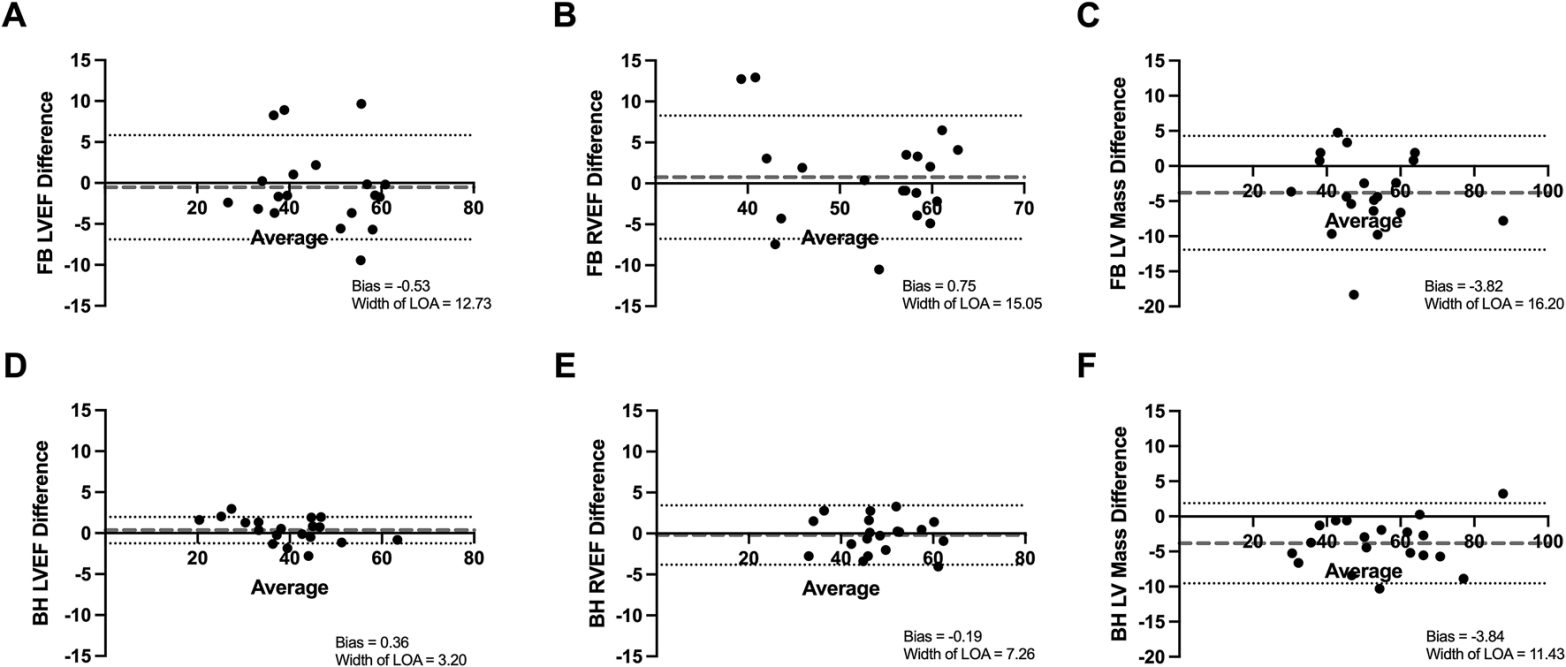
Intra-observer Bland–Altman plots. (**A**) Assessment of LVEF in FB scans. (**B**) Assessment of RVEF in FB scans. (**C**) Assessment of LV Mass in FB scans. (**D**) Assessment of LVEF in BH scans. (**E**) Assessment of RVEF in BH scans. (**F**) Assessment of LV mass in BH scans. *BH* breath-held scan, *FB* free-breathing scan, *LV* left ventricle, *LVEF* left ventricle ejection fraction, *LOA* limit of agreement, *RVEF* right ventricle ejection fraction.

Correlation scatter and Bland–Altman plots (Figs. 2, 3) show clearly improved linear associations, tighter grouping of data points and reduced bias in the BH cohort.

### Inter-observer variability

Similarly improved reproducibly indices were observed on analysis of variability between two independent observers (Table 3). Reduced coefficients of variation, mean absolute differences and Bland–Altman limits of agreements were all observed in the BH cohort.

**Table 3:** Inter-observer variability.

Parameter	Modality	Correlation co-efficient (r)	Mean absolute difference	SD of difference	CV (%)	Width of BA-LOA
LVEF	FB	0.79	5.78	7.25	10.82	28.42
	BH	0.97	2.17	2.63	4.49	10.32
RVEF	FB	0.78	8.63	15.03	8.86	27.4
	BH	0.96	2.07	2.47	3.41	9.67

*BA-LOA* Bland–Altman limit of agreement, *BH* breath-held, *CV* co-efficient of variation, *FB* free-breathing, *LVEF* left ventricular ejection fraction, *RVEF* right ventricular ejection fraction, *SD* standard deviation.

**Figure 4:**
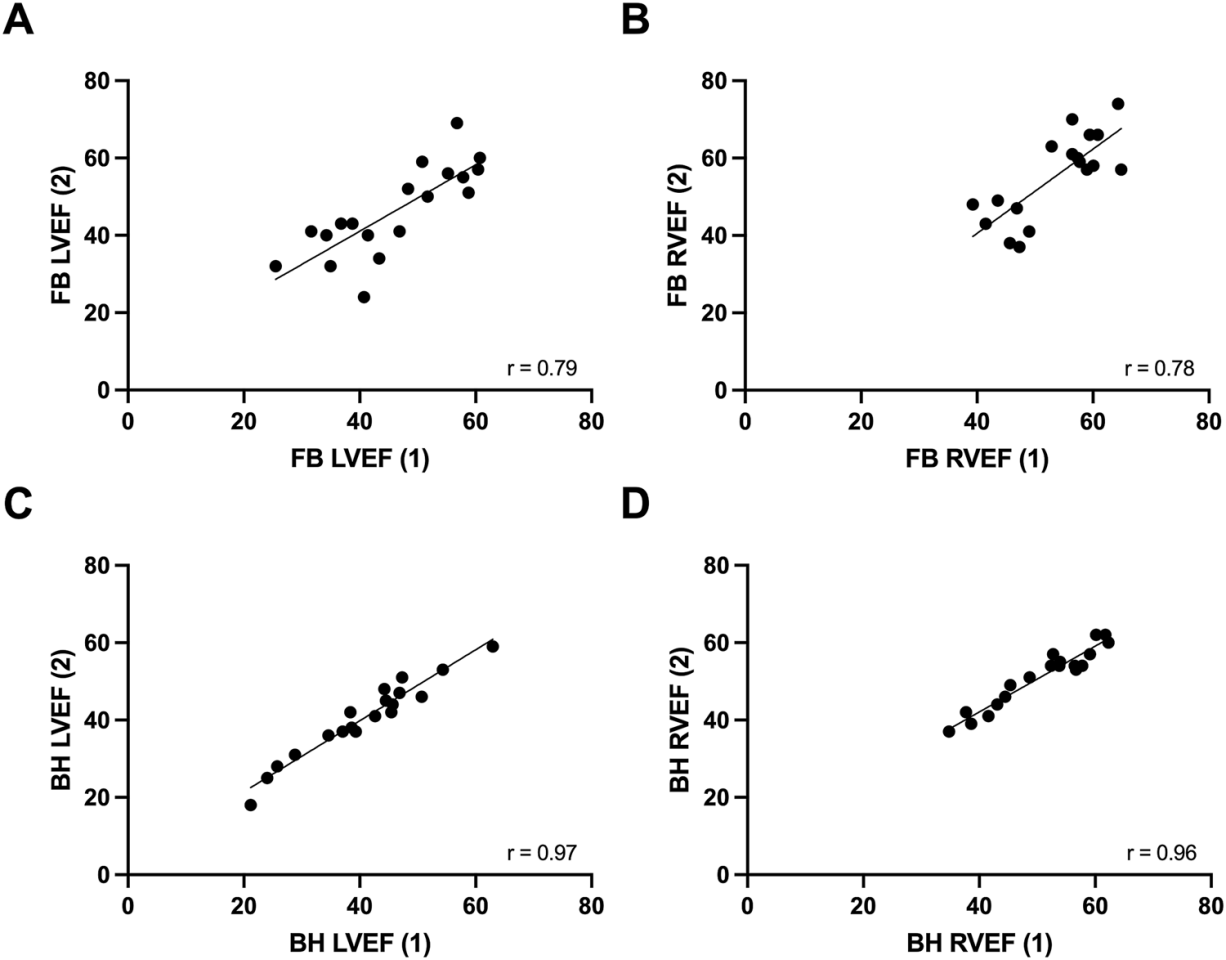
Inter-observer correlation scatter plots. (**A**) Assessment of LVEF in FB scans. (**B**) Assessment of RVEF in FB scans. (**C**) Assessment of LVEF in BH scans. (**D**) Assessment of RVEF in BH scans. (1) = Observer 1; (2) = Observer 2. *BH* breath-held scan, *FB* free-breathing scan, *LVEF* left ventricle ejection fraction, *r* Pearson’s correlation co-efficient, *RVEF* right ventricle ejection fraction.

**Figure 5:**
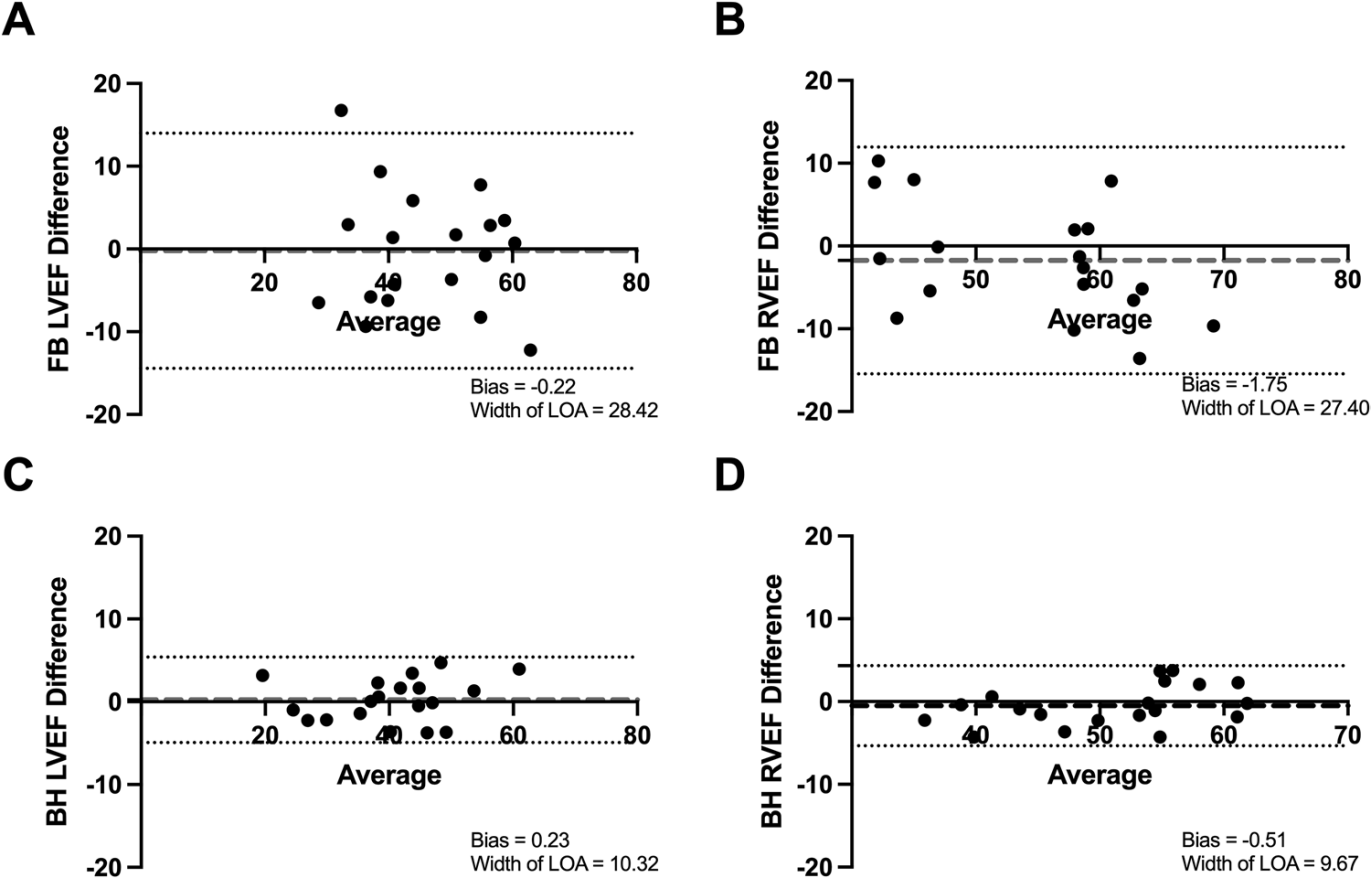
Inter-observer Bland–Altman plots. (**A**) Assessment of LVEF in FB scans. (**B**) Assessment of RVEF in FB scans. (**C**) Assessment of LVEF in BH scans. (**D**) Assessment of RVEF in BH scans. *BH* breath-held scan, *FB* free-breathing scan, *LVEF* left ventricle ejection fraction, *LOA* limit of agreement, *RVEF* right ventricle ejection fraction.

Correlation scatter and Bland–Altman plots (Figs. 4, 5) also demonstrated reduced bias and improved linear association.

### Sample size calculations

The smaller inter-observer SD of difference in BH subjects translated to substantially lower sample sizes required by BH compared to FB CMR to detect a clinically significant difference in left or right ventricular ejection fraction (Table 4). To detect an absolute LVEF change of 5%, a total of 44 subjects per group would be required if undergoing FB image acquisition, as opposed to only 6 subjects per group with BH scans. This accounts for an 86% reduction in sample size by BH acquisition. Even more substantially, to detect a 5% absolute change in RVEF a 97% reduction in group sizes can be achieved, with required sample sizes reducing from 190 subjects to only 5.

**Table 4:** Sample sizes required to detect clinically significant changes in ejection fraction.

	Free-breathing CMR		Breath-held CMR		Reduction in sample size by breath held CMR (%)
	SD	Sample size (n)	SD	Sample size (n)	
5% absolute change in LVEF	7.3	44	2.6	6	86.4
5% absolute change in RVEF	15.0	190	2.5	5	97.4

*CMR* cardiac magnetic resonance, *LVEF* left ventricle ejection fraction, *RVEF* right ventricle ejection fraction, *SD* inter-observer pooled standard deviation.

Sample sizes calculated with a 90% power and an $\mathrm{\alpha }$ error of 0.05.

## Discussion

Quantification of biventricular volume and ejection fraction is a critical endpoint in the pre-clinical evaluation of novel cardio-protective agents^2^, and it is imperative that researchers have confidence in the interpretation and analysis of acquired data. Even though CMR is considered the gold standard of non-invasive cardiac volumetric assessment^4–6^, there exists equipoise with respect to the best protocol for CMR cine analysis in pre-clinical studies, with both FB^11–15^ and BH^17–20^ protocols being utilized. In this study we have shown that substantial variability may exist even with CMR depending on the method used to acquire images, translating to significant animal welfare issues.

Retrospective ECG-gated cine CMR with breath-hold is the standard CMR protocol for assessment of LV volumes in human patients^21,22^. However, free-breathing has emerged as an attractive alternative^10,11,23,24^, liberating patients and clinicians from breath-hold constraints. The nature of large animal research dictates that animals be fully anaesthetised and mechanically ventilated to facilitate BH CMR^25^. However, factors such as research costs, availability of specialist equipment, and lack of personnel trained in veterinary anaesthetic care and recovery, make free-breathing scans with comparatively ‘simpler’ sedation strategies an attractive option. In addition, maintenance inhalant anaesthesia can cause a degree of vasodilation, hypotension and myocardial depression^26,27^; factors which may confound results and increase procedural risk in a myocardial infarction model.

By retrospectively analysing 38 consecutive porcine CMRs conducted by our group, in which there was equal distribution of FB and BH scans, we have shown that:

1. Breath-held porcine CMR produces superior image quality as compared to FB CMR in both subjective and objective assessments along with shorter scan acquisition.
2. There is greater intra- and inter-observer reproducibility in assessment of common CMR metrics with BH scans.
3. Undertaking BH CMR can result in substantially reduced sample sizes required to show clinically significant treatment effects.

To our knowledge, we are the first group to report reproducibility data of FB and BH CMR in a large animal cohort.

### Breath-held CMR produces superior image quality and shorter scan acquisition

Breath-held CMR resulted in significant improvement in subjective image quality as assessed by two independent observers blinded to group allocation. The majority of BH scans were rated as having above average image quality, as assessed by clarity of endocardial borders and ease of volumetric contouring. Only one BH scan was rated as having below acceptable image quality, and on review it was noted that this scan was complicated by an arrhythmia at the time of image acquisition, an independent variable which contributes to CMR artefact^7^. This is despite the absence of a neuromuscular blockade agent in our anaesthetic protocol, which may have further improved image quality. Free-breathing scans on the other hand were largely rated as having poor image quality. Respiratory motion artefact is well known to complicate CMR acquisition^7,28,29^, and this study confirms this phenomenon plays an important role in free-breathing porcine subjects.

Image quality was also objectively assessed by quantifying image intensity in myocardium and blood pool for both LV and RV chambers. This ratio was shown to be greater in BH scans, confirming crisper delineation at the tissue: blood interface in BH scans, a critical factor for accurate contouring^16^.

In addition, given that only one rather than three signal averages were required for the BH cohort, time taken to acquire the cine short axis stack was significantly shorter compared to FB subjects (4:06 ± 1:55 vs. 8:53 ± 2:39; *p* < 0.0001), resulting in reduced time under anaesthesia; an important animal welfare consideration.

### Breath-held CMR has greater reproducibility

Superior image quality translated to improved reproducibility of volumetric indices and myocardial mass measurements. This was particularly apparent when assessing the right ventricle, in which the improvement in reproducibility by breath-holding was especially striking. The inter-study reproducibility of right ventricular volumes is generally thought to be similar to, or only slightly lower than, the much more well studied left ventricle^6,30,31^. However, previous RV reproducibility studies employed breath-held acquisitions, and here we show that the right ventricle is particularly susceptible to the motion artefact conferred by free-breathing, likely owing to the chamber’s thin walls and complex geometry^32,33^.

### Breath held CMR results in reduced sample sizes

The impact of interstudy reproducibility can truly be appreciated when considering power and sample size calculations based on the SD_diff_ between repeated studies. Greater reproducibility (low SD_diff_) increases the reliability of observed treatment effects, reducing sample sizes required to show statistical significance^5^. This has powerful implications in study design for pre-clinical trials, and we sought to use this as a metric to compare each CMR method. Sample size is related to the SD_diff_ by a square function, thus, even small improvements in reproducibility between methods can have greatly magnified effects on sample size. Here we show that BH acquisition can reduce animal numbers approaching and exceeding 90% when compared with FB.

Though there may be greater setup costs associated with equipment and personnel required to facilitate BH CMR, this may be offset by the reduced animal numbers needed to show significant treatment effects and faster study completion. In addition, and perhaps more importantly, animal welfare is a priority in scientific research. The ‘Three Rs’ tenet—replacement, reduction and refinement^34^, should be a cornerstone principle in all laboratories conducting translational research, with our study advocating for this ethos.

### Study limitations

Due to the expense and resources involved in conducting large animal experiments, only 19 subjects in each group could be studied. Though our study was still able to show substantial difference between modalities, increased subject numbers would have improved power, and studying chronic infarct subjects may also have been of interest with our investigation of acute and sub-acute infarcts perhaps not extrapolatable to this cohort.”.

Additionally, free-breathing CMR protocols to mitigate motion artefact through synchronization or compensation now exist, however must be purchased at additional cost and were unavailable to us at the time of conducting this study. Comparing breath-held CMR to a motion compensated or synchronized free-breathing protocol will be worthwhile for future studies, along with conducting cost analyses for each method.

Furthermore, each subject should have ideally undergone both free-breathing and breath-held image acquisition, allowing for fair internal comparisons between modalities. Unfortunately, though this may be an entirely feasible strategy in human patients, it is considerably more difficult both logistically and practically with porcine subjects, requiring more complex anaesthetic strategies, and increasing scan time which may result in overheating or run the risk of exceeding specific absorption rate (SAR) limits.

Finally, all images were acquired prior to the administration of gadolinium contrast. It may be of interest to also compare methods in a post-contrast dataset, in which contour detection may be further impaired due to a reduction in the myocardium:blood pool intensity.

## Conclusion

Accurate and reproducible assessment of intracardiac volumes is of paramount importance in the preclinical evaluation of novel cardioprotective or regenerative agents. Breath-held CMR, although associated with greater initial setup costs and veterinary anaesthesia expertise, results in superior image quality, shorter scan acquisition, greater reproducibility and reduced sample sizes required to show clinically significant treatment effects.

## Methods

### Study population

We retrospectively examined CMR data from porcine subjects enrolled into study protocols investigating the utility of novel agents for ischaemic heart failure. Details and efficacy of these agents are not discussed here, as the aims of this study were solely to examine the quality and reproducibility of porcine CMR acquired from either a FB or BH acquisition. There was a similar distribution of treated and control subjects between the FB and BH groups (Table 1). All experiments were conducted in accordance with local guidelines and regulations, and all study protocols were approved by the Animal Ethics Committee of the Western Sydney Local Health District. Authors complied with the ARRIVE guidelines^3^ for conducting animal research.

Experiments were conducted in 38 adult, female Landrace swine (Table 1) which were housed individually and receiving standard care. All animals were acquired at approximately 2 months of age from the same local supplier and acclimatised to the housing facility for at least 1 week before commencing experimental procedures.

### Myocardial Infarction

Myocardial infarction was percutaneously induced by methods previously described by our group^35^. Briefly, animals were premedicated with intramuscular ketamine (10 mg/kg), methadone (0.3 mg/kg), and midazolam (0.3 mg/kg), intubated, ventilated, and maintained with inhalational isoflurane. The left coronary artery was engaged percutaneously via the right femoral artery using a 6F hockey stick guiding catheter (Medtronic, Minnesota, U.S.A). A 0.36-mm coronary guidewire (Sion Blue, Asahi Intecc Co. Ltd., Aichi, Japan) was delivered into the left anterior descending artery (LAD). Myocardial infarction was induced by occluding the mid LAD distal to the first diagonal branch with an inflated 2.0–3.0-mm angioplasty balloon (Boston Scientific, Massachusetts, U.S.A) for 90 min. Coronary angiography performed after reperfusion confirmed vessel patency and resolution of ST elevation. Ventricular arrythmias were treated with anti-arrhythmics and defibrillation as required.

### Cardiac magnetic resonance imaging

CMR was performed post induction of myocardial infarction (16.2 ± 12.2 days) according to either a FB, or BH sedation and scan protocol as described below. The first 19 subjects in our study underwent a FB CMR acquisition protocol, and following an institutional policy change, the subsequent 19 subjects underwent a BH protocol.

#### Free-breathing scans

Animals were premedicated with intramuscular ketamine (10 mg/kg), methadone (0.3 mg/kg), and midazolam (0.3 mg/kg) before intubation to maintain airway protection. Mechanical ventilation was not employed, and animals were allowed to breathe freely throughout the duration of the scan. Sedation depth was periodically monitored by clinically assessing for animal movement, rate and depth of breathing, muscle tone, and heart rate. Top-up sedation was administered as required with propofol or midazolam boluses.

#### Breath-held scans

Animals were premedicated with intramuscular ketamine (10 mg/kg), methadone (0.3 mg/kg), and midazolam (0.3 mg/kg), before intubation and mechanical ventilation. General anesthesia was induced with intravenous propofol (2–5 mg/kg) and maintained with 2% inhaled isoflurane. Ventilatory parameters were adjusted to maintain an end-tidal CO_2_ of 35–45 mmHg. Anaesthetic depth was periodically monitored by clinically assessing for animal movement, eye position, loss of jaw tone, and absence of palpebral and pedal reflexes. Breathing was held in end-expiration for all image acquisitions.

#### CMR protocol

All CMR examinations were performed on a Siemens 3T Prisma system (Siemens Medical Systems, Erlangen, Germany) utilising an 18-channel body array together with spine array coils and 4-lead electrocardiogram (ECG) gating. All images for this study were acquired before the addition of gadolinium contrast. Axial and coronal TrueFISP (true fast imaging with steady state precession) sequences through the heart were acquired to plan preliminary 2-chamber, short-axis, and 4-chamber single-slice gradient images. True 2-chamber, 3-chamber, 4-chamber, and right ventricular outflow tract 8 mm single-slice TRUFI (true fast imaging) cines were then planned and acquired, as well as a short-axis stack of 14 contiguous 8 mm slices, starting just beyond the apex and extending into the atrium, planned from 2-and 4-chamber cine images in end-diastole.

TrueFISP imaging was used for all acquisitions with the following parameters: TE (echo time) 1.3 ms, TR (repetition time) at R-R interval of individual animal, FOV (field-of-view) 370 mm, slice thickness 8 mm, in-plane resolution 1.4 mm × 1.4 mm, flip angle 10 degrees, 25 calculated phases. One signal average was acquired for breath-hold imaging with the acquisition time being around 10 s whereas three signal averages were collected for free-breathing acquisitions to reduce motion artifact. For the short-axis free-breathing sequence, all 14 slices were acquired in a single run whereas for breath-hold acquisitions 3–5 slices were acquired during each breath-hold of around 30 s (depending on R-R interval), resulting in 3–5 breath-holds per sequence.

#### Image analysis

**Figure 6:**
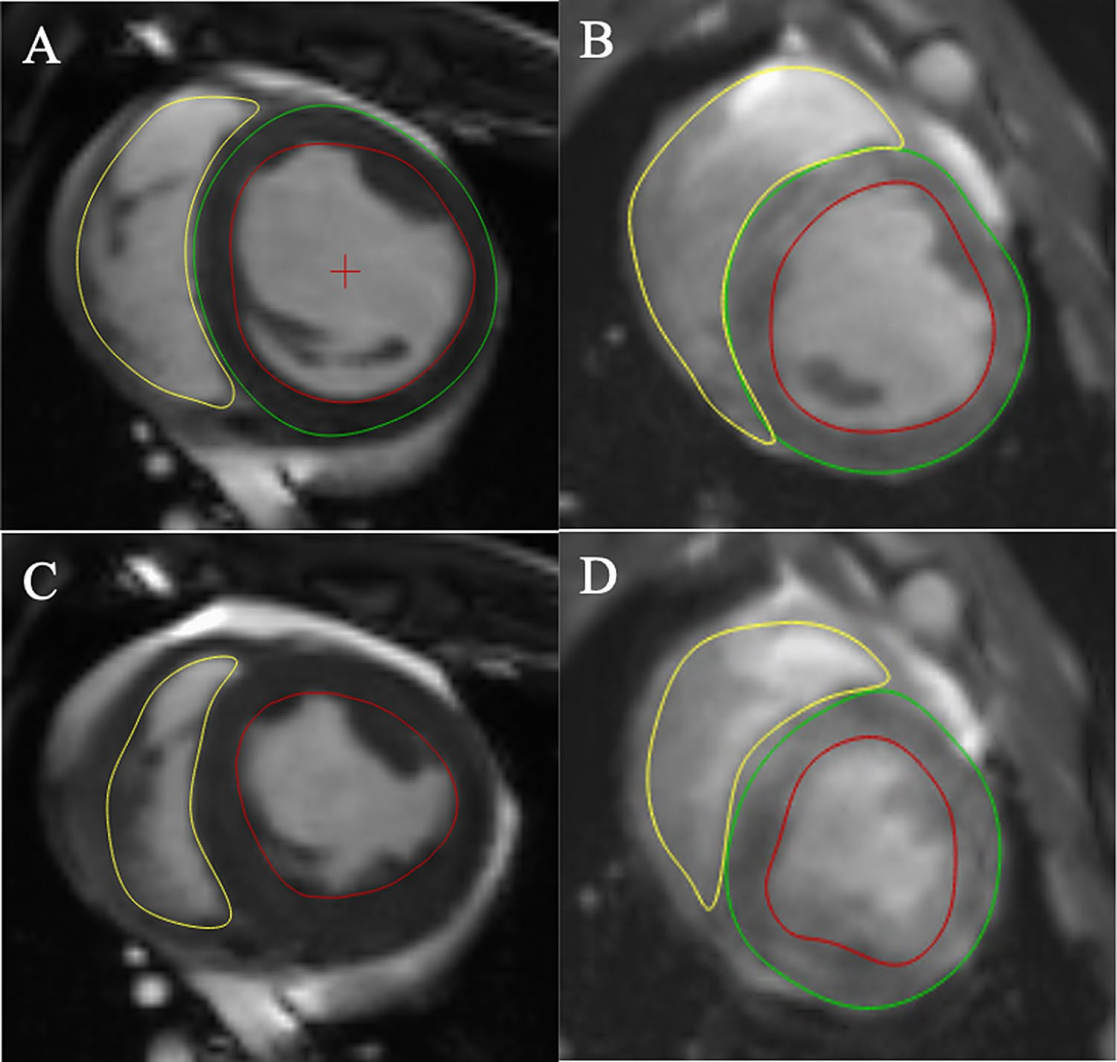
Representative images and contouring of breath-held and free-breathing scans. (**A**) Breath-held scan in end-diastole. (**B**) Free-breathing scan in end-diastole. (**C**) Breath held scan in end-systole. (**D**) Free-breathing scan in end-systole.

To assess reproducibility between approaches, left and right ventricular (LV and RV) end-diastolic volume (EDV), end-systolic volume (ESV) and ejection fraction (EF) were calculated by two independent, blinded observers. Intra-observer variability was determined by having one observer, blinded to previous measurements, repeat measurements on all subjects on two separate occasions, at least two weeks apart. Image analysis was performed offline using dedicated software (Medis Suite MR 3.2, Medis Medical Imaging, Schuttersveld 9, The Netherlands). Volumetric and myocardial mass assessment were performed according to the Society for Cardiovascular Magnetic Resonance guidelines^36^ (Fig. 6). In brief, short axis end-diastolic and end-systolic images were chosen as the maximal and minimal mid-ventricular cross-sectional areas. End-diastolic epicardial and endocardial borders, along with end-systolic endocardial borders were manually traced for each slice. The vendor specific automated contouring algorithm was not used due to suboptimal performance in non-human subjects. Papillary muscles were included in volume and excluded in mass calculations. The difference between end-diastolic and end-systolic endocardial borders represented the left or right ventricular stroke volume, and ejection fraction was calculated as the stroke volume/end-diastolic volume. Myocardial volume was derived from slice thickness along with the difference between left ventricular end-diastolic epicardial and endocardial volumes. Mass (g) was derived from this volume multiplied by the specific density of myocardium (1.05 g/cm^2^).

Image quality was assessed using 2 methods:

1. Edge discrimination was qualitatively scored on a scale of 1–5 based on the clarity of the endocardial borders and ease of volumetric analysis (1 = uninterpretable, 2 = poor, 3 = acceptable, 4 = good, 5 = very good). Scoring was performed by the same two independent observers for all subjects. In cases with differing scores, the case was re-adjudicated by both observers until a consensus was reached.
2. Contrast quality was objectively assessed by quantifying LV and RV blood pool and myocardial image intensity at the mid-ventricular level. Regions of interest (ROIs) were drawn on the LV septum and RV free wall and the signal intensity measured. Separately ROIs measured signal intensity in left and right ventricular blood pools. The respective LV:blood pool and RV:blood pool signal intensity ratios were used as a marker of image quality which accurately allows discrimination of the myocardium from the blood pool^16^.

#### Statistical analysis

Statistical analyses were performed using IBM SPSS Statistics version 26 (IBM Corporation, New York, U.S.A) and GraphPad Prism version 9.0.1 (GraphPad Software, San Diego, U.S.A). Parametric data were summarized by the mean ± standard deviation (SD). Independent sample t tests or Mann–Whitney U tests were performed depending on distribution of data, and significance was set as an α < 0.05.

Inter- and intra-observer variability was expressed using Pearson’s correlation coefficient (r) to quantify the strength of linear association between repeated measurements. The coefficient of variation (CV) and mean absolute difference were additionally used to assess agreement between repeated measurements. CV (expressed as a percentage) was calculated as the intra-observer pooled standard deviation divided by the mean of the parameter under consideration. Bland–Altman plots were used to illustrate each pairwise agreement, showing the systematic bias and limits of agreement (mean difference ± 2 × SD_diff_, where SD_diff_ is the SD of differences).

#### Sample size calculation

The sample sizes required to show a clinical change with a power of 90% and an error of 0.05 were calculated using the following formula^5,37^:

$$ n = f\left( {\upalpha ,{\text{P}}} \right)*\sigma^{2} *\frac{2}{{d^{2} }} $$

where $$n$$ is the sample size, $$\upalpha $$ the significance level, P the study power required and $$f$$ the value of the factor for different values of $$\upalpha $$ and P ($$f $$ = 10.5 for $$\upalpha $$ = 0.05 and P = 0.90), with $$\sigma$$ the inter-observer pooled SD_diff_ and *d* the desired difference to be detected.

## Data Availability

The datasets generated and analysed during the current study are available from the corresponding author on reasonable request.
